# Endoscopic transorbital approach to recurrent clinoidal meningioma

**DOI:** 10.3171/2025.10.FOCVID25171

**Published:** 2026-01-01

**Authors:** Francesco Paglia, Lorenzo Sgarbanti, Ben Chat-Fong Ng, Carlo Conti, Hunter Kwok-Lai Yuen, Calvin Hoi-Kwan Mak

**Affiliations:** 1 Human Neurosciences Department, University La Sapienza, Rome, Italy;; 2 Department of Translational Medicine, Neurosurgery Unit, University of Ferrara, Ferrara, Italy;; 3 Department of Neurosurgery, Queen Elizabeth Hospital, Hong Kong;; 4 Department of Neurosurgery, Azienda Ospedaliera S.Maria di Terni, Terni, Italy; and; 5 Hong Kong Eye Hospital Department of Ophthalmology and Visual Sciences, The Chinese University of Hong Kong, Hong Kong

**Keywords:** transorbital endoscopic approach, sagittal crest, meningo-orbital band, anterior clinoid

## Abstract

Anterior clinoid meningiomas are challenging lesions due to their proximity to critical neurovascular structures. Traditional surgical approaches often require extensive bone removal and brain retraction, increasing the risk of complications. The transorbital endoscopic approach has emerged as a novel, minimally invasive alternative, offering direct access to the anterior cranial base and parasellar region through the orbital cavity, minimizing manipulation of brain tissue while maintaining effective tumor resection. Current evidence supports its feasibility and safety in selected skull base cases. The authors present the case of a 66-year-old woman with a recurrent atypical anterior clinoidal meningioma previously treated with a frontotemporal craniotomy.

The video can be found here: https://stream.cadmore.media/r10.3171/2025.10.FOCVID25171

## Transcript

This video shows an endoscopic transorbital approach for resection of a recurrent atypical clinoidal meningioma.

### 0:27 Clinical Presentation and Neurological Examination.

A 66-year-old female with a history of recurrent intracranial meningioma previously treated in 2016 with transcranial microsurgical resection of a WHO grade II sphenoidal ridge meningioma via a left pterional approach, followed by adjuvant intensity-modulated radiation therapy. At radiological follow-up in November 2024, MRI demonstrated tumor progression involving the left anterior clinoid process. Clinical evaluation showed mild left-sided proptosis, right homonymous hemianopia, and decreased left visual acuity.

### 1:01 Neuroimaging and Choice of Approach.

MRI showed a left clinoidal meningioma compressing the ipsilateral optic nerve. Due to the proximity to the second cranial nerve, radiotherapy was not indicated. Preoperative cranial CT confirmed a regular morphology of the left clinoid without pneumatization or anatomical variants such as a caroticoclinoid foramen. To achieve a Simpson grade I resection, an endoscopic transorbital approach was selected. This minimally invasive route, increasingly adopted for the management of complex skull base tumors,^1^ provides a direct trajectory to the anterior clinoid process while avoiding brain retraction. Moreover, it minimizes the cosmetic and functional morbidity often associated with frontotemporal craniotomy, such as temporalis muscle atrophy and masticatory pain.^2^

### 1:51 Positioning and Surgical Equipment.

The procedure was performed under general anesthesia with the patient in a supine neutral position and the head secured in a Mayfield skull clamp. Magnetic neuronavigation with navigated suction was used. The initial phase was conducted using an exoscope, followed by an endoscope.

### 2:10 Skin Incision.

Through a subbrow incision, subperiosteal dissection of the periorbita was performed up to the superior orbital fissure. The orbital rim was flattened with a coarse diamond burr under periorbital protection, exposing the temporal fascia. Drilling was then redirected to the greater sphenoid wing until the temporal dura was reached.

### 2:30 Subperiosteal Dissection of the Periorbita and Meningo-Orbital Band.

The sagittal crest, a nonnative anatomical landmark, was exposed and progressively removed.^3,4^ The meningo-orbital band was dissected with endoscopic scissors. A 0° endoscope was introduced to complete crest removal, expose the anterior cranial fossa dura, and finalize the meningo-orbital band incision.

### 2:55 Anterior Clinoidectomy.

Using a 2-mm Kerrison rongeur, superior orbital fissure is opened, and as shown in this anatomical picture, using an eggshell technique, anterior clinoid process is progressively entered using a 2-mm pure diamond drill, alternating drilling and suction. Magnetic neuronavigation provides to confirm the correct trajectory. Finally, using a rongeur, the tip of the anterior clinoid process is gently dissected and removed, taking care to avoid injury of the adjacent neurovascular structures.^5^ Hemostatic agents are used to stop venous bleeding after anterior clinoidectomy. Anterior cranial fossa dura is progressively exposed and dura mater cutting is started in a lateromedial direction.

### 3:52 Meningioma Removal.

Using the arachnoid plane and alternating gentle traction and cutting of the arachnoid bridge, the almost completely devascularized meningioma is removed en bloc avoiding brain manipulation. After inspection of the surgical field, the pieces of the tumor adherent to the brain are removed and hemostasis is completed using Surgicel. In order to achieve a Simpson grade I resection, the dura mater is cut and removed.

### 4:28 Reconstruction.

The small dural defect is repaired in a multilayered fashion. Reconstruction begins with an inlay of dura patch reinforced by fibrin glue. Another dura patch is used to isolate the surgical field. Valsalva maneuver is performed in order to confirm the absence of cerebrospinal fluid leak.

### 4:50 Postoperative Neuroimaging and Postoperative Clinical Outcome.

The patient tolerated the procedure well, with no new neurological deficits, and was discharged on postoperative day 2. Postoperative CT showed no complications, and 1-year follow-up MRI confirmed absence of recurrences.

### 5:09 Limitations of ETOA.

The main limitations of the transorbital approach include tumor exceeding 6 cm in diameter, predominant invasion of the frontal lobe, and/or extension to the supraclinoid segment of the internal carotid artery.

## Disclosures

The authors report no conflict of interest concerning the materials or methods used in this study or the findings specified in this publication.

## Author Contributions

Primary surgeon: Mak. Assistant surgeon: Yuen. Editing and drafting the video and abstract: Paglia, Sgarbanti, Ng. Critically revising the work: Mak, Paglia, Sgarbanti, Conti. Reviewed submitted version of the work: Mak, Paglia, Sgarbanti, Yuen. Approved the final version of the work on behalf of all authors: Mak. Supervision: Mak.

## Supplemental Information

### Patient Informed Consent

The necessary patient informed consent was obtained in this study.
